# No effect modification of serum bilirubin or coffee consumption on the association of gamma-glutamyltransferase with glycated hemoglobin in a cross-sectional study of Japanese men and women

**DOI:** 10.1186/1472-6823-12-24

**Published:** 2012-10-23

**Authors:** Zhenjie Wang, Christopher McMonagle, Shinichiro Yoshimitsu, Sanjeev Budhathoki, Makiko Morita, Kengo Toyomura, Keizo Ohnaka, Ryoichi Takayanagi, Suminori Kono

**Affiliations:** 1 Department of Preventive Medicine, Graduate School of Medical Sciences, Kyushu University, Fukuoka, 812-8582, Japan; 2 MRC Epidemiology Unit, Institute of Metabolic Science, Addenbrooke’s Hospital, Cambridge, UK; 3 Department of Geriatric Medicine, Graduate School of Medical Sciences, Kyushu University, Fukuoka, Japan; 4 Department of Medicine and Bioregulatory Science, Graduate School of Medical Sciences, Kyushu University, Fukuoka, Japan

## Abstract

**Background:**

Oxidative stress has been implicated in the development of type 2 diabetes mellitus. Bilirubin is a potent endogenous antioxidant, and coffee is a major source of exogenous antioxidants. Serum gamma-glutamyltransferase (GGT), a marker of oxidative stress, is a strong predictor of the risk of type 2 diabetes mellitus. This study evaluated the effect modification of bilirubin and coffee consumption on the association of serum GGT with glycated hemoglobin (HbA1c) and the combined effect of bilirubin and coffee on HbA1c concentrations.

**Methods:**

The subjects were 4492 men and 6242 women aged 49–76 years who participated in the baseline survey of an on-going cohort study on lifestyle-related diseases in Fukuoka, Japan. Geometric means of HbA1c were examined according to quartile categories of GGT, with stratification by serum total bilirubin (≥ 0.6 mg/dL *versus* less in men and ≥ 0.5 mg/dL *versus* less in women) and coffee consumption (< 1, 1–3 and ≥ 4 cups of per day). Statistical adjustment was made for age, smoking, alcohol use and body mass index by using analysis of covariance.

**Results:**

HbA1 concentrations increased progressively with increasing levels of GGT in both men and women. The increasing trend of HbA1c concentrations associated with GGT did not differ by either bilirubin status or coffee consumption. Both men and women with high bilirubin had consistently lower concentrations of HbA1c across the GGT quartiles. Higher coffee consumption was associated with lower concentrations of HbA1c in women with low bilirubin (trend *P* = 0.04), but not with high bilirubin (trend *P* = 0.37). There was no such association between coffee and HbA1c in men with either low or high bilirubin levels.

**Conclusions:**

Bilirubin is possibly protective against deterioration of glucose metabolism. Further studies are needed regarding the combined effect of bilirubin and coffee on glucose metabolism.

## Background

Oxidative stress has been implicated in the development of type 2 diabetes mellitus [1,2]. *In vitro* and animal studies showed that antioxidants improved insulin sensitivity and were protective against beta-cell injury [3,4]. Bilirubin, the end product of heme catabolism, is a potent endogenous antioxidant [5-7]. Enhancement of bilirubin formation by induction of heme oxygenase-1 ameliorated glucose metabolism and insulin sensitivity in rodents [8,9]. High bilirubin concentrations were inversely related to the prevalence of type 2 diabetes mellitus [10,11] and of the metabolic syndrome [12]. On the other hand, coffee is a major source of exogenous antioxidants in populations in which coffee is commonly consumed [13-15]. Coffee drinking has consistently been shown to be protective against type 2 diabetes mellitus and glucose intolerance [16,17].

Elevated activities of serum gamma-glutamyltransferase (GGT) are shown to be a strong predictor of type 2 diabetes mellitus independent of obesity index [18]. While hepatic steatosis associated with visceral adiposity may be a common mediator for elevated GGT and insulin resistance [19], GGT may be a marker of oxidative stress [20,21]. GGT is a plasma membrane enzyme involved in the so-called gamma-glutamyl cycle by which extracellular glutathione is transported into cells for the synthesis of intracellular glutathione. Depletion of intracellular antioxidant glutathione in response to oxidative stress results in an increase in GGT so that the redox regulation is maintained. Interestingly, it was shown that a decreased risk of type 2 diabetes associated with coffee consumption was more pronounced in individuals with elevated activities of serum GGT [22]. It is thus hypothesized that a protective effect of antioxidants against type 2 diabetes mellitus or glucose metabolism may depend on the magnitude of the background oxidative stress.

In the study reported here, we evaluated the effect modification of serum bilirubin and coffee intake on the association between GGT and glycated hemoglobin (HbA1c) in Japanese men and women. The combined effect of bilirubin and coffee on HbA1c concentrations was a matter of another interest.

## Methods

### Study subjects

The subjects were participants in the baseline survey of an on-going cohort study on lifestyle-related diseases. The study was approved by the Ethics Committee of the Faculty of Medical Sciences, Kyushu University. All the subjects gave written informed consent. A detailed description of the baseline survey has been provided elsewhere [10]. Briefly, residents aged 50–74 years in the East Ward of Fukuoka City were invited to the survey by mail, and a total of 12948 persons participated in the survey from February 2004 to August 2007, with a participation rate of 24%. Age at the survey ranged 49 to 76 years; three women aged 49 years were included because of a mistake in recording the date of birth.

Stored serum for the measurement of serum bilirubin was available for 12942 participants. We excluded 2195 subjects for the following reasons: medication for diabetes mellitus (*n* = 680); current medical care for coronary artery diseases (*n* = 533), stroke (*n* = 276), cancer (*n* = 496) or chronic hepatitis or cirrhosis (*n* = 232); prior history of myocardial infarction or coronary angioplasty (*n* = 40) or stroke (*n* = 152); aspartate aminotransferase (AST) or alanine aminotransferase (ALT) greater than 3-fold of the upper limit of the normal range, i.e., >120 U/L (*n* = 59); GGT greater than 3-fold of the upper limit of the normal range, i.e., > 450 U/L (*n* = 43); total bilirubin > 3.0 mg/dL (*n* = 3); and serum creatinine > 2.0 mg/dL (*n* = 44). The cutoff values for liver enzymes, bilirubin and creatinine were defined arbitrarily, but were aimed to exclude those with pathological conditions which possibly affected lifestyle factors and/or glucose metabolism. Further excluded were 13 subjects who had missing values for liver enzymes, creatinine, alcohol use or body mass index. Finally, 10734 subjects (4492 men and 6242 women) remained in the present analysis.

### Laboratory measurements

Non-fasting or fasting venous blood samples were taken for the determination of HbA1c and serum bilirubin concentrations. Only 470 subjects (4.4%) had been fasting for 10 hours or longer. Serum was separated by centrifugation on the same day and frozen at −80°C. Whole-blood samples for the measurement of HbA1c were transported to an external laboratory (SRL, Tokyo) on the next working day after a temporary storage in refrigerator. HbA1c was determined by using the latex agglutination immunoassay [23]. Total bilirubin concentrations were measured at the above-described laboratory by the absorptiometric assay with oxidation by vanadic acid [24]. Regarding serum GGT, AST, ALT, creatinine and other 4 biochemical measurements, recorded information was referred to at the survey if they had been determined within the past one year. Otherwise, 5 mL of venous blood was taken, and the measurements were carried out at the same laboratory as described above. The recorded measurements were available for 3924 subjects (36.6%), and the measurements were done at the survey for the remaining 6810 (63.4%). In the former group, 3191 (81.3%) reported that the measurements was done after an overnight fast; 319 (4.7%) in the latter were in a fast of 10 hours or longer at the survey.

### Lifestyle questionnaire

Dietary and non-dietary lifestyle factors, diseases under medical treatment, history of selected diseases and other factors were assessed by a self-administered questionnaire with supplementary interview. The food frequency questionnaire developed by Tokudome et al. [25] was modified with respect to alcohol and beverage consumption. Alcohol users were defined as those who had consumed alcoholic beverages at least once per week over a period of one year or more, and former alcohol drinkers were distinguished from lifelong abstainers. Current alcohol drinkers reported consumption frequencies and amounts of five types of alcoholic beverages on average over the past year. Likewise, habitual consumption over the past one year was ascertained regarding coffee and other beverages, by using closed-ended questions with eight options (none, 1–2, 3–4 or 5–6 cups per week and 1–3, 4–6, 7–9 or ≥10 cups per day). Smokers were defined as those who had smoked one or more cigarettes per day for at least one year, and former smokers were separated from life-long non-smokers. Former and current smokers reported the average number of cigarettes smoked per day and the total number of years of smoking.

### Anthropometric measurements

Height (cm) and weight (kg) were measured, and body mass index was calculated as weight divided by the square of height (kg/m^2^).

### Statistical analysis

All analyses were performed in men and women separately. GGT levels were categorized by using sex-specific quartiles. Individuals were classified into high (≥ 0.6 mg/dl in men and ≥ 0.5 mg/dl in women) and low bilirubin groups. These cutoff points appeared to be a threshold in the sex-specific relationship with HbA1c levels [10], and corresponded to the gender-wise medians. Coffee consumption was categorized into < 1, 1–3 and ≥ 4 cups of coffee per day. The following covariates were always taken into account: age (continuous), smoking (never, past and current smoking with a consumption of < 20 or ≥ 20 cigarettes/day), alcohol drinking (never, past and current drinking with a consumption < 30, 30–59 or ≥ 60 mL of ethanol/day) and body mass index (sex-specific quartiles). Trends of the associations of GGT levels and coffee consumption with HbA1c were evaluated by assigning ordinal values to GGT and coffee categories. Effect modification of bilirubin or coffee on the association between GGT and HbA1c was evaluated by using a product term of the ordinal variable for GGT and a variable for bilirubin (dichotomous) or coffee (three categories). Statistical significance was declared if two-sided *P* was less than 0.05. Statistical analyses were performed by SAS version 9.2 (SAS Institute, Cary, NC).

## Results

Selected characteristics of the study subjects are summarized in Table 1. Women had lower values of body mass index and GGT than men. Bilirubin concentrations were slightly lower in women than in men, as indicated by a lower value of the upper quartile while HbA1c levels were higher in women. Geometric means of HbA1c increased progressively with increasing levels of GGT in both men and women. In men, age-adjusted geometric means of HbA1c (%) for the quartile categories of GGT (≤ 23, 24–35, 36–58 and ≥ 59 IU/L) were 5.00, 5.08, 5.16 and 5.20 respectively (trend *P* < 10^–15^), and multivariate-adjusted geometric means were 4.99, 5.07, 5.16 and 5.23 respectively (trend *P* < 10^–18^). In women, age-adjusted geometric means of HbA1c (%) according to the quartile categories (≤ 15, 16–20, 21–29 and ≥ 30 IU/L) were 5.00, 5.06, 5.13 and 5.17 respectively (trend *P* < 10^–32^), and multivariate-adjusted geometric means were 5.00, 5.07, 5.12 and 5.16 respectively (trend P < 10^–26^).

**Table 1:** Selected characteristics of the study subjects by sex

**Variable**	**Men (*n*= 4492)**	**Women (*n*= 6242)**
Age (year), mean (SD)	62.3 (6.8)	61.9 (6.7)
Body mass index (kg/m^2^), mean (SD)	23.5 (2.7)	22.5 (3.1)
Smoking (cigarettes/day), %		
Never	26.1	88.7
Former	42.4	5.2
< 20	9.2	3.7
≥ 20	23.3	2.4
Alcohol use (mL/day), %		
Never	21.1	70.4
Former	5.9	2.4
< 30	29.6	23.5
30–59	26.2	3.0
≥ 60	17.1	0.8
Daily use of coffee, %	57.5	58.0
Serum GGT (IU/L), median (IQR)	35 (24–58)	20 (16–29)
Serum total bilirubin (mg/dL), median (IQR)	0.5 (0.4–0.7)	0.5 (0.4–0.6)
HbA1c (%), median (IQR)	5.0 (4.8–5.3)	5.1 (4.8–5.3)

GGT, gamma-glutamyl transferase; IQR, interquartile range; SD, standard deviation.

The increasing trend of HbA1c concentrations associated with GGT did not differ by either bilirubin status or coffee consumption (Table 2). Both men and women with high bilirubin had consistently lower concentrations of HbA1c across the GGT quartiles. With adjustment for GGT (quartile category) and the other covariates, the geometric means of HbA1c (%) for the low and high bilirubin levels were 5.15 (95% CI 5.12–5.17) and 5.07 (95% CI 5.05–5.10) respectively in men (*P* < 10^–4^), and 5.12 (95% CI 5.11–5.14) and 5.06 (95% CI 5.04–5.07) respectively in women (*P* < 10^–9^). The adjusted geometric means of HbA1c for coffee categories of < 1, 1–3 and ≥ 4 cups per day were 5.11 (95% CI 5.08–5.14), 5.09 (95% CI 5.07–5.12) and 5.18 (95% CI 5.13–5.24) respectively in men (trend *P* = 0.20) and 5.09 (95% CI 5.08–5.11), 5.09 (95% CI 5.07–5.10) and 5.06 (95% CI 5.03–5.10) respectively in women (trend *P* = 0.18).

**Table 2:** Adjusted geometric means of HbA1c for quartiles of gamma-glutamyltransferase (GGT) by bilirubin and coffee consumption*

		**GGT†**				**Trend**	**Interaction**
**Stratification**	***n***	**Q1 (lowest)**	**Q2**	**Q3**	**Q4 (highest)**	***P*‡**	***P***
**Men**							
Bilirubin (mg/dL)							
Low (< 0.6)	2313	5.02 (4.97–5.07)	5.11 (5.06–5.16)	5.21 (5.16–5.26)	5.26 (5.20–5.31)	< 10^–9^	0.72
High (≥ 0.6)	2179	4.95 (4.89–5.00)	5.02 (4.97–5.07)	5.12 (5.07–5.17)	5.20 (5.15–5.25)	< 10^–10^	
Coffee (cups/day)							
< 1	1911	5.00 (4.95–5.06)	5.07 (5.02–5.13)	5.14 (5.09–5.20)	5.24 (5.18–5.29)	< 10^–6^	0.64
1–3	2036	4.96 (4.91–5.01)	5.05 (5.00–5.10)	5.16 (5.11–5.21)	5.20 (5.14–5.26)	< 10^–9^	
≥ 4	545	5.03 (4.94–5.12)	5.13 (5.03–5.23)	5.27 (5.17–5.37)	5.32 (5.19–5.45)	< 10^–3^	
**Women**							
Bilirubin (mg/dL)							
Low (< 0.5)	2910	5.02 (4.99–5.05)	5.10 (5.07–5.13)	5.18 (5.15–5.21)	5.19 (5.16–5.22)	< 10^–16^	0.22
High (≥ 0.5)	3332	4.99 (4.96–5.02)	5.03 (5.01–5.06)	5.07 (5.04–5.10)	5.14 (5.11–5.17)	< 10^–12^	
Coffee (cups/day)							
< 1	2623	5.02 (4.99–5.06)	5.06 (5.03–5.10)	5.12 (5.09–5.15)	5.17 (5.14–5.20)	< 10^–9^	0.86
1–3	3081	4.99 (4.96–5.02)	5.07 (5.05–5.10)	5.13 (5.10–5.16)	5.15 (5.12–5.18)	< 10^–15^	
≥ 4	538	4.99 (4.93–5.06)	5.02 (4.95–5.09)	5.08 (5.01–5.15)	5.17 (5.09–5.24)	< 10^–3^	

In parentheses are 95% confidence intervals of geometric means.

* Adjusted for age, smoking, alcohol intake and body mass index by analysis of covariance.

† The first to the fourth quartile (Q1 to Q4) categories were ≤ 23, 24–35, 36–58 and ≥ 59 IU/L in men; and ≤ 15, 16–20, 21–29 and ≥ 30 IU/L in women.

‡ Ordinal values were assigned to GGT categories.

HbA1c concentrations according to the combination of bilirubin and coffee use are shown in Table 3. High bilirubin was consistently associated with lower concentrations of HbA1c regardless of coffee consumption except for men consuming ≥ 4 cups of coffee per day. Higher coffee consumption was associated with lower concentrations of HbA1c in women with low bilirubin (trend *P* = 0.04), but not with high bilirubin (trend *P* = 0.37), while the interaction between bilirubin and coffee was far from the statistical significance. In men, HbA1c concentrations tended to be lower only among those consuming 1–3 cups of coffee per day. Serum GGT was weakly positively correlated with bilirubin concentrations in men (Spearman *r* = 0.099), and there was no correlation between the two in women (Spearman *r* = −0.004). With further adjustment for GGT (quartile category), the results for the combined categories of bilirubin and coffee did not change appreciably, while the decreasing trend of HbA1c with coffee consumption in women with low bilirubin failed to reach the statistical significance (trend *P* = 0.06).

**Table 3:** Adjusted geometric means of HbA1c according to combined categories of bilirubin and coffee consumption*

		**Low bilirubin†**		**High bilirubin†**	
**Sex**	**Coffee (cups/day)**	***n***	**Geometric mean**	***n***	**Geometric mean**
Men	< 1	877	5.16 (5.12–5.21)	1034	5.08 (5.05–5.12)
	1–3	1077	5.13 (5.09–5.16)	959	5.04 (5.01–5.08)
	≥ 4	359	5.16 (5.10–5.23)	186	5.18 (5.10–5.27)
	Trend‡		*P* = 0.74		*P* = 0.55
Women	< 1	1125	5.14 (5.12–5.17)	1498	5.06 (5.04–5.08)
	1–3	1503	5.11 (5.09–5.13)	1578	5.06 (5.04–5.08)
	≥ 4	282	5.09 (5.04–5.14)	256	5.02 (4.97–5.07)
	Trend‡		*P* = 0.04		*P* = 0.37

In parentheses are 95% confidence intervals of geometric means. Interaction *P* values were 0.43 in men and 0.37 in women.

* Adjusted for age, smoking, alcohol intake and body mass index by analysis of covariance.

† Low: < 0.6 mg/dL in men and < 0.5 mg/dL in women; high: ≥ 0.6 mg/dL in men and ≥ 0.5 mg/dL in women.

‡ Ordinal values were assigned to coffee categories.

## Discussion

Expectedly, GGT levels were strongly, positively associated with HbA1c concentrations in both men and women. The present study showed no effect modification of either bilirubin or coffee consumption on the association between GGT and HbA1c. High bilirubin was consistently associated with lower HbA1c concentrations regardless of GGT levels.

The inverse association between serum bilirubin and HbA1c itself is not a novel finding [10,11]. We previously reported an inverse association between bilirubin and HbA1c in the present study population [10]. It was also shown among dyslipidemic patients that bilirubin concentrations were lower and serum GGT was higher in patients with the metabolic syndrome as compared with those without [12]. Nonetheless, it is notable that HbA1c was consistently lowered in those with high bilirubin across GGT levels. A cross-sectional inverse association of bilirubin with HbA1c or type 2 diabetes mellitus does not necessarily indicate a causal relationship because hyperglycemia itself may decrease serum bilirubin due to elevated oxidative stress [26]. The present findings add to evidence that bilirubin is protective against deterioration of glucose metabolism. Both bilirubin and GGT levels increase in the presence of hepatobiliary diseases, but bilirubin and GGT are generally uncorrelated with each other in healthy subjects [27]. In the present study population, bilirubin was weakly correlated with GGT only in men.

In addition to a possible protective effect against type 2 diabetes mellitus [16,17], coffee has consistently been shown to be associated with lower activities of serum GGT [22]. Indeed, coffee consumption was strongly, inversely associated with serum GGT in the present study population as well [28]. Coffee did not affect the association between GGT and HbA1c in either men or women, and there was no tendency that coffee drinkers had lower HbA1c concentrations when their GGT levels were higher. The present findings are seemingly in disagreement with the previous observation that a decreased risk of type 2 diabetes mellitus associated with coffee consumption was more marked in those with higher levels of GGT [22]. However, the present study did not address the association between coffee and the risk of type 2 diabetes mellitus, and the findings do not necessarily exclude the possibility that the risk of type 2 diabetes is decreased with coffee consumption in high normal GGT levels.

The present study firstly addressed the association between bilirubin and coffee in combination and HbA1c concentrations. None has addressed the combined effect of bilirubin and coffee on the risk of type 2 diabetes. Because coffee and bilirubin are potent antioxidants, it can be hypothesized that coffee’s effect on glucose metabolism may be modified by bilirubin status and *vice versa*. The inverse association between coffee consumption and HbA1c observed among women with low bilirubin is compatible with this hypothesis, but no such association was observed in men. Careful interpretation is needed, however, because the analysis on the combination of bilirubin and coffee included four statistical tests for the trend (Table 3). There is a 15% chance that at least one of the four *P* values will be 0.04 or less even if there is no association between coffee and HbA1c. The combined effect of bilirubin and coffee on glucose metabolism would deserve further studies. It should be noted that coffee may exert a protective effect on glucose metabolism via mechanisms other than the antioxidant effect. *In vitro* studies have suggested that antioxidant coffee compounds such as chlorogenic acid and caffeic acid delay glucose absorption in the intestine, inhibit glucose output in the liver and increase peripheral glucose uptake [29].

Although men consuming 1–3 cups of coffee per day tended to have slightly lower concentrations of HbA1c, those with the highest coffee consumption had rather elevated HbA1c concentrations, especially when their bilirubin was high. We have no prompt explanation for the latter finding. The finding may be a chance fluctuation because the number of such men was small, and alternatively may be ascribed to residual confounding factors which were not controlled for in the present analysis.

In addition to a fairly large size of the study, some advantages of the present study are noted. HbA1c and bilirubin concentrations were uniformly measured, and important confounders were taken into account. Several weaknesses should be mentioned, however. Self-reported coffee consumption suffers inaccuracy, causing measurement misclassification. Causal inference is difficult for the cross-sectional association, as discussed above. Comorbid conditions may affect serum bilirubin concentrations and coffee consumption. Therefore, we excluded participants who had life-limiting morbid conditions such as atherosclerotic disease, cancer and liver disease. A low level of participation (24%) in the survey is of another concern in interpreting the results. Finally, it should be noted that the measurement in the past year was used for GGT among one-third of the subjects. Those having high GGT levels before the survey may have been under treatment for diabetes mellitus or prediabetic condition, and thereby distorting the association between GGT and HbA1c. In fact, the association between GGT and HbA1c was slightly weaker among men, not among women, with the past measurement of GGT than among men without. The adjusted geometric means of HbA1c (%) for the above-mentioned quartile categories of GGT were 5.00, 5.05, 5.14 and 5.15 respectively in men with the recorded measurement (trend *P* < 10^–5^) and 4.98, 5.08, 5.18 and 5.28 respectively in men with GGT measurement at the survey (trend *P* < 10^–14^). However, the analysis of the subjects with GGT measurement at the survey showed almost the same results as reported above with respect to the associations with bilirubin and coffee and the effect modifications of these covariates (data not shown).

## Conclusions

In a large cross-sectional study of Japanese men and women, a high level of serum bilirubin was consistently associated with lower HbA1c concentrations across the GGT levels. Coffee consumption was associated with lower HbA1 concentrations in women, but not in men, with a low bilirubin status. Bilirubin is possibly protective against deterioration of glucose metabolism. Further studies are needed regarding the combined effect of bilirubin and coffee on glucose metabolism.

## Abbreviations

AST: Aspartate aminotransferase; ALT: Alanine aminotransferase; GGT: Gamma-glutamyltransferase; HbA1c: Glycated hemoglobin; IQR: Interquartile range; SD: Standard deviation.

## Competing interests

None of the authors had either financial or non-financial competing interests to be declared.

## Authors’ contributions

ZW, CM, SY and SB were in charge of the development of the concept, statistical analysis and interpretation of the results, and prepared the first draft of the manuscript. MM, KT and KO contributed to acquisition and compilation of the data and revision of the manuscript. RT and SK were in charge of the design and implementation of the survey and contributed to revision of the manuscript. All authors approved the final report.

## Pre-publication history

The pre-publication history for this paper can be accessed here:

http://www.biomedcentral.com/1472-6823/12/24/prepub
